# Burnout Assessment Tool for Students (BAT-S): evidence of validity in a Chilean sample of undergraduate university students

**DOI:** 10.3389/fpsyg.2024.1434412

**Published:** 2024-12-18

**Authors:** Marcos Carmona-Halty, Karina Alarcón-Castillo, Carla Semir-González, Geraldy Sepúlveda-Páez, Wilmar B. Schaufeli

**Affiliations:** ^1^Escuela de Psicología y Filosofía, Universidad de Tarapacá, Arica, Chile; ^2^Department of Psychology, Utrecht University, Utrecht, Netherlands; ^3^Research Unit Occupational & Organizational Psychology and Professional Learning, KU Leuven, Leuven, Belgium

**Keywords:** burnout assessment tool, psychometric analysis, undergraduate students, burnout, academic burnout

## Abstract

This brief report examines both within-network and between-network construct validity of the Burnout Assessment Tool for Students (BAT-S) in a sample of 461 Chilean undergraduate university students (70.9% female) ranging between 18 and 58 years old (*M* = 21.6, SD = 4.34). The reliability analysis results showed adequate internal consistency for the overall burnout score and for each dimension. In addition, confirmatory factor analysis (CFA) supported a second-order factor (academic burnout) and four first-order factors (exhaustion, mental distance, cognitive impairment, and emotional impairment) solution. Moreover, the results of multiple-group CFA supported gender invariance. Finally, structural equation model (SEM) analysis showed that academic resources and academic demands are associated with academic burnout. Overall, the BAT-S was found to be a reliable and valid tool to assess academic burnout in chilean sample of undergraduate university students.

## Introduction

Burnout is a metaphor that refers to a state of work-related mental exhaustion (Maslach and Jackson, 1981; Maslach et al., 2001; Schaufeli et al., 2020). However, it can also be used in relation to all activities that are structured, coercive in nature and are oriented toward achieving specific goals, such as those performed by students (Schaufeli and Taris, 2005). Following this line of reasoning, academic burnout traditionally describes those students who are mentally exhausted, have a cynical and detached attitude toward their studies, and feel incompetent as students (Schaufeli et al., 2002).

The current literature shows that academic burnout is directly related to study-related negative emotions (Carmona-Halty et al., 2022), study holism (Sanseverino et al., 2023), intention to drop out of school (Marôco et al., 2020), and anxiety (Popescu et al., 2023). Conversely, it is inversely related to engagement (Wang et al., 2021), self-efficacy (Kong et al., 2021), well-being (Yu and Chae, 2020), and achievement (Madigan and Curran, 2021). Furthermore, based on the application of the Job Demands Resources (JD-R) model (Bakker and Demerouti, 2017) in the academic context, academic demands (e.g., study overload) and academic resources (e.g., teacher support), promote and prevent its occurrence, respectively (Lesener et al., 2020; Salmela-Aro et al., 2022; Salmela-Aro and Upadyaya, 2014; Zeijen et al., 2024).

Research on academic burnout has mostly been conducted using the Maslach Burnout Inventory-Student Survey (MBI-SS) developed by Schaufeli et al. (2002). The MBI-SS is an adaptation of the Maslach Burnout Inventory General Survey (MBI-GS; Maslach et al., 1997) and has been widely used in both samples of high school students and undergraduate university students (e.g., Madigan and Curran, 2021; Salanova et al., 2010; Salmela-Aro et al., 2022; Vizoso et al., 2019; Xie et al., 2019). Despite the relevance that the MBI-SS has had for the study of burnout in academic settings, the conceptual, psychometric, and practical weaknesses of the MBI-GS –given their equivalencies– can be reasonably generalized to the use of the MBI-SS (for a systematic and meta-analytical review, see De Beer et al., 2024).

Addressing the limitations of the MBI-GS, Schaufeli et al. (2020) developed the Burnout Assessment Tool (BAT), a new tool for individual and group assessment of burnout. For this purpose, they conducted interviews with 50 health professionals –who attended to burned-out people on a daily basis– using a dialectical method with deductive and inductive approaches. The content analysis of the interviews revealed four core dimensions: exhaustion, mental distance, cognitive impairment, and emotional impairment.

From this perspective, academic burnout describes those students who experience a severe loss of energy that results in feelings of both physical and mental fatigue (i.e., being exhausted); a strong reluctance or aversion to study, indifference, and cynicism (i.e., being mentally distanced); memory problems, attention and concentration deficits, and poor cognitive performance (i.e., cognitive impairment); and intense emotional reactions such as anger or sadness and feeling overwhelmed by one’s emotions (i.e., emotional impairment).

In this new conceptualization, exhaustion plays a central role in reducing the capacity to regulate cognitive and emotional processes and their subsequent deterioration. At the same time, mental distance is considered a counterproductive coping strategy that contributes to the increase in exhaustion (Schaufeli and De Witte, 2023). Consequently, students who experience high levels of burnout have problems processing information and managing their emotions. In an attempt to cope with these issues, they distance themselves psychologically from their stressful academic activities, leads to negative consequences (e.g., non-fulfillment of commitments, problems with peers, accumulation of academic load, poor academic performance), which, in their turn aggravate feeling of stress and burnout.

On the one hand, the BAT produces a composite score, and, on the other hand also scores for each of the four symptom-dimensions. Hence, it has a hierarchical structure equivalent to a model of four first-order factors (i.e., exhaustion, mental distance, cognitive impairment, and emotional impairment) and one higher-order factor (i.e., burnout), which is consistent with the notion of a burnout syndrome (World Health Organization, 2019). Its psychometric properties, both of the original (BAT-23), the short (BAT-12) and ultra-short (BAT-4) versions, have been demonstrated in various countries (e.g., Italy–Consiglio et al., 2021; Croatia–Tomas et al., 2023; South Africa–De Beer et al., 2022a; Greece–Androulakis et al., 2023; Norway–De Beer et al., 2023; Romania–Oprea et al., 2021; Japan–Sakakibara et al., 2020; Australia–Redelinghuys and Morgan, 2023; Equator–Vinueza-Solórzano et al., 2021; Brazil–Sinval et al., 2022; among others–Basinka et al., 2021; De Beer et al., 2020; Hadžibajramović et al., 2022, 2024). In addition, different language versions (e.g., Italian, Japanese, French, and Spanish) and a student version (BAT-S) are currently available.

Despite the increasingly robust body of research generated around the validity of the BAT, psychometric analysis of this tool in an academic context is still scarce (for a review, see Schaufeli and De Witte, 2023). So far, only two studies have been published that have demonstrated the psychometric properties of the BAT-S to date. First, Romano et al. (2022), in a sample of 745 students from two Italian public middle schools, report that the structure of four first-order factors (i.e., exhaustion, mental distance, cognitive impairment, and emotional impairment) and 1 second-order factor (i.e., academic burnout) fits significantly better compared to a series of alternative models (e.g., a unidimensional model). Additionally, the authors report that both the composite and dimension scores are significantly related to well-being, resilience, anxiety, and exhaustion indicators. Second, Popescu et al. (2023), in a sample of 399 Romanian undergraduate students, support the second-order factor structure and describe significant relationships with indicators of depression, anxiety, stress, psychosomatic symptoms, prospective evaluation of future tasks, and coping strategies. Hence, it seems relevant to continue investigating the psychometric properties of the BAT-S, also in other national and cultural contexts.

The current research is unique as it aims to provide the first validation of the student version of the BAT in a Spanish-speaking context. So, this study fills a gap by examining the psychometric properties of the short 12-item version of the BAT-S in a sample of Chilean undergraduate students following both within-network and between-network construct validity. The first refers to assessing reliability, factor structure, and gender invariance, while the second refers to assessing the extent to which academic burnout is associated with theoretically related constructs. More specifically, we use as a conceptual framework the Job Demands Resources (JD-R) model (Bakker and Demerouti, 2017), which is one of the most applied frameworks in occupational health psychology for examining the relationship between employee well-being and its antecedents and outcomes (Bakker and Demerouti, 2017; De Beer et al., 2022b; Schaufeli and Taris, 2014), and has been successfully applied in the academic context (e.g., Salmela-Aro et al., 2022; Salmela-Aro and Upadyaya, 2014; Zeijen et al., 2024). In this line, academic demands can be defined as the aspects of the studies that require sustained effort and are associated with certain physiological and psychological costs, while academic resources can be defined as the aspects of the studies that have motivating potential, that are functional in achieving work goals, that regulate the impact of academic demands, and that stimulate learning and personal growth (Bakker et al., 2023). In the present study we focus on study overload and teacher support, two constructs that have previously been considered as academic demand and resource and have been shown to be related to academic burnout (Lesener et al., 2020; Salmela-Aro et al., 2022; Zeijen et al., 2024).

Based on the background information presented, our hypotheses are as follows: (1) the abbreviated version of BAT-S will demonstrate acceptable psychometric properties in a sample of Chilean undergraduate university students; (2) academic demands will be positively associated with academic burnout; and (3) academic resources will be negatively associated with academic burnout.

## Methods

### Sample

The initial sample consisted of 474 Chilean undergraduate students. Following the recommendations of the literature on careless responding (e.g., Ward and Meade, 2023), the final sample consisted of 461 Chilean undergraduate students from the following programs: health (52.4%; *n* = 241), social sciences (38.1%; *n* = 176), engineering (5.4%; *n* = 25), and education (4.1%; *n* = 19). Of the total participants, 70.9% (*n* = 327) identified themselves as female and 29.1% (*n* = 134) as male, with an age range between 18 and 58 years (*M* = 22.4; SD = 4.34).

### Instruments

The abbreviated version of the BAT-S was used (available at)^1^. This version includes 12 items that assess –using a Likert-type response format with scores between 1 (*never*) and 5 (*always*)– the four dimensions of academic burnout: exhaustion (3 items, e.g., “*Due to my studies, I feel mentally exhausted*”), mental distance (3 items; e.g., “*I struggle to find any enthusiasm for my studies*”), cognitive impairment (3 items; e.g., “*When I am working on my studies, I have trouble staying focused*”), and emotional impairment (3 items; e.g., “*I feel unable to control my emotions*”). The adaptation to the usual conditions of the Chilean undergraduate students was carried out following the guidelines of the International Test Commission (2017) and the specialized literature (see Muñiz et al., 2013; Vallejo-Medina et al., 2017). Prior to the data collection, the items were evaluated in a pilot study by a sample of undergraduate Chilean students (*n* = 10) who were asked to point out any difficulties associated with the comprehension of the items and the response format. At this stage, no student expressed problems with the wording of the items or with the item response format.

The teacher-student relationship scale (Martin et al., 2007) was used to measure teacher support (which is considered an academic resource). This scale has 4 items (e.g., *“My teachers give me the help and support I need”*) and a Likert-type response format was used with scores ranging from 1 (*strongly disagree*) to 7 (*strongly agree*). Adequate Cronbach alpha (*α* = 0.899) and McDonald’s omega (*ω* = 0.901) indices were obtained in the present study. As a measure of study overload (considered an academic demand), we use a self-constructed six-item scale (e.g., *“Currently, I have a heavy academic workload”*) that assesses the perception of academic overload using a Likert-type response format, with scores ranging from 1 (strongly disagree) to 7 (strongly agree). Adequate Cronbach alpha (*α* = 0.909) and McDonald’s omega (*ω* = 0.910) indices were obtained in the present study.

### Procedure

The data were collected in the context of a research project that sought to analyze the well-being levels of the Chilean university population. The project was approved by the research ethics committee of the host university. Participants voluntarily completed an online questionnaire during their regular class hours. The time taken to answer the questionnaire was approximately 15 min.

### Statistical analyses

All analyses were performed with JASP (2021) v 0.18.3 and Mplus v 8.2 (Muthén and Muthén, 1998) software. First, the distribution characteristics of the variables were analyzed (mean, standard deviation, skewness, kurtosis, and Shapiro–Wilk test), as well as gender differences (independent *t*-tests). Second, the internal structure of the BAT-S was analyzed by performing a confirmatory factor analysis (CFA) with a weighted least square mean and variance-adjusted (WLSMV) extraction method. The goodness of fit was assessed by calculating the chi-square (*χ*2) and normalized χ2, the root mean square error of approximation (RMSEA) with a 90% confidence interval (CI), the comparative fit index (CFI) and the standardized root mean residual (SRMR). The global fit indicators of the models were interpreted according to the guidelines proposed by Hair et al. (2019). Third, the reliability of the scores was estimated with the Cronbach’s alpha and McDonald’s omega indexes with 95% confidence interval (CI). Fourth, to establish the equivalence of the BAT-S between students’ gender, a second-order multiple-group CFA was performed following the recommendations of Wang and Wang (2019). Changes in CFI of 0.010 or less (Chen, 2007; Cheung and Rensvold, 2002; Dimitrov, 2010) were considered a criterion for determining whether measurement invariance was established. Fourth, to examine criterion validity, a structural equation model (SEM) was performed to evaluate the role of academic demands and resources in academic burnout, assessed through BAT-S.

## Results

### Descriptive analysis

Table 1 shows the descriptive statistics of the Spanish version of BAT-S at the item level. The Shapiro–Wilk test showed that the items are not normally distributed. Independent-sample *t*-tests revealed that –in accordance with meta-analytical studies (e.g., Purvanova and Muros, 2010; Fiorilli et al., 2022)– female students (*M* = 2.959, SD = 0.694) scored significantly higher than male (*M* = 2.808, SD = 0.741) students, *t* (459) = 2.085, *p* < 0.050, *d* = 0.214, 95% CI (0.012, 0.415). However, the effect size is small based on Cohen’s (1988) criterion.

**Table 1:** Descriptive and reliability information at item level of BAT-S and factor loading resulting from confirmatory factor analysis.

	Descriptive statistics				Reliability statistics			Factor loadings				
	M (SD)	S	K	SW	ω if item is dropped	α if item is dropped	CHI	EX	MD	CI	EI	SE
1. Due to my studies, I feel mentally exhausted	3.776 (0.894)	-0.472	-0.042	0.872*	0.862	0.858	0.601	0.862*				0.025
2. After a day of working on my study, I find it hard to recover my energy	3.466 (1.115)	-0.223	-0.851	0.900*	0.863	0.860	0.575	0.812*				0.024
3. While working on my studies, I feel physically exhausted	3.504 (1.063)	-0.287	-0.537	0.901*	0.865	0.862	0.543	0.811*				0.022
4. I struggle to find any enthusiasm for my studies	3.133 (1.189)	0.006	-0.843	0.912*	0.863	0.859	0.610		0.802*			0.027
5. I feel a strong aversion toward my studies	2.487 (1.090)	0.347	-0.442	0.896*	0.863	0.859	0.620		0.820*			0.029
6. I’m cynical about what my study means to others	2.013 (1.141)	0.978	0.154	0.809*	0.881	0.881	0.239		0.361*			0.050
7. When I am working on my studies, I have trouble staying focused	3.169 (1.102)	0.099	-0.733	0.908*	0.861	0.857	0.623			0.870*		0.019
8. When I am working on my studies. I have trouble concentrating	3.468 (1.042)	-0.118	-0.755	0.900*	0.865	0.862	0.542			0.794*		0.022
9. I make mistakes while working on my studies because I have my mind on other things	3.019 (1.118)	0.154	-0.854	0.907*	0.862	0.859	0.591			0.752*		0.025
10. I feel unable to control my emotions	2.502 (1.171)	0.475	-0.632	0.893*	0.860	0.857	0.633				0.844*	0.023
11. I do not recognize myself in the way I react emotionally	2.308 (1.142)	0.547	-0.556	0.877*	0.862	0.859	0.610				0.824*	0.022
12. I may overreact unintentionally.	2.278 (1.198)	0.657	-0.544	0.862*	0.867	0.863	0.527				0.696*	0.030

* *p* < 0.001; M, mean; SD, standard deviation; S, skewness; K, kurtosis; SW, Shapiro–Wilk test; CHI, corrected homogeneity index; EX, exhaustion; MD, mental distance; CI, cognitive impairment; EI, emotional impairment; SE, standard error.

### Internal structure

Two models were specified to evaluate the internal structure of the Spanish version of the BAT-S. The first model (M1) assumes that one latent factor is underlying all scale items, whereas Model 2 (M2) proposes a structure of four first-order factors (i.e., exhaustion, mental distance, cognitive impairment, and emotional impairment) and 1 second-order factor (i.e., academic burnout). The results show that the one-factor solution does not obtain adequate fit indices and, therefore, is not a good representation of the data collected (M1 in Table 2), while the second-order factor solution obtains adequate fit indices except for the RMSEA value (M2 in Table 2). Therefore, we examined the modification indices and proceeded to covary the measurement error of items 7 and 9, which both refer to the difficulty in staying focused and correspond to the cognitive impairment dimension (see Table 1). As a result, the re-specified second-order factor solution (M4 in Table 2) demonstrates an adequate fit to the data. Table 1 shows the factor loadings obtained for the M4.

**Table 2:** Fit indexes for the single-group and multiple-group CFA of the BAT-S.

	*X* ^2^	*df*	*p*	*X*^2^/*df*	RMSEA	90% CI	CFI	TLI	SRMR	CMs	ΔCFI
Single-group CFA											
M1 one factor	895.061	54	0.000	16.575	0.184	[0.173, 0.194]	0.827	0.789	0.082	-	-
M2 second order	218.124	50	0.000	5.297	0.085	[0.074, 0.097]	0.965	0.954	0.039	-	-
M3 one factor re-specified	894.876	53	0.000	16.884	0.186	[0.175, 0.196]	0.827	0.784	0.081	-	-
M4 second order re-specified	170.296	49	0.000	3.475	0.073	[0.061, 0.085]	0.975	0.966	0.035	-	-
Multiple-group CFA											
M5 Configural invariance	121.147	94	0.031	1.288	0.035	[0.011, 0.052]	0.982	0.975	0.040	-	-
M6 Metric invariance	128.033	102	0.041	1.255	0.033	[0.007, 0.050]	0.983	0.978	0.044	M5-M6	0.001
M7 Scalar invariance	152.290	110	0.004	1.384	0.041	[0.023, 0.056]	0.973	0.967	0.050	M6-M7	0.010
M8 Configural invariance *	128.961	102	0.036	1.264	0.034	[0.009, 0.051]	0.983	0.977	0.042	-	-
M9 Metric invariance *	134.773	109	0.047	1.236	0.032	[0.004, 0.049]	0.983	0.980	0.047	M8-M9	0.000

*, second order invariance; *χ*2, Chi-square; df, degree of freedom; RMSEA, root mean square error of approximation; 90% CI, confidence interval; CFI, comparative fit index.

TLI, Tucker-Lewis index; SRMR, standardized root mean square residual; CMs, comparisons between models.

### Reliability of the scores

The Spanish version of BAT-S, based on Kalkbrenner (2021), shows adequate internal consistency both for the global score (*ω* = 0.874, 95% CI [0.856, 0.891]; *α* = 0.870, 95% CI [0.852, 0.887]) and for each of its dimensions: exhaustion (*ω* = 0.828, 95% CI [0.800, 0.855]; *α* = 0.823, 95% CI [0.794, 0.849]), mental distance (*ω* = 0.689, 95% CI [0.640, 0.738]; *α* = 0.652, 95% CI [0.593, 0.704]), cognitive impairment (*ω* = 0.804, 95% CI [0.773, 0.835]; *α* = 0.798, 95% CI [0.763, 0.828]), and emotional impairment (*ω* = 0.795, 95% CI [0.762, 0.827]; *α* = 0.792, 95% CI [0.757, 0.823]).

### Measurement invariance

A second-order multiple-group CFA was performed to assess whether the structure of the BAT-S is equivalent according to the gender of the students. Following Wang and Wang (2019), the first step was to verify the configural invariance of the second-order model (M8 in Table 2). Next, three levels of equivalence (i.e., configural, metric, scalar) of the first-order factors were verified (M5, M6, M7 in Table 2). Finally, the metric invariance of the second-order model was verified (M9 in Table 2). All model fits were adequate, and the differences in the CFI met the established criteria, supporting the equivalence of the second-order structure regarding student gender.

### Criterion validity

**Figure 1:**
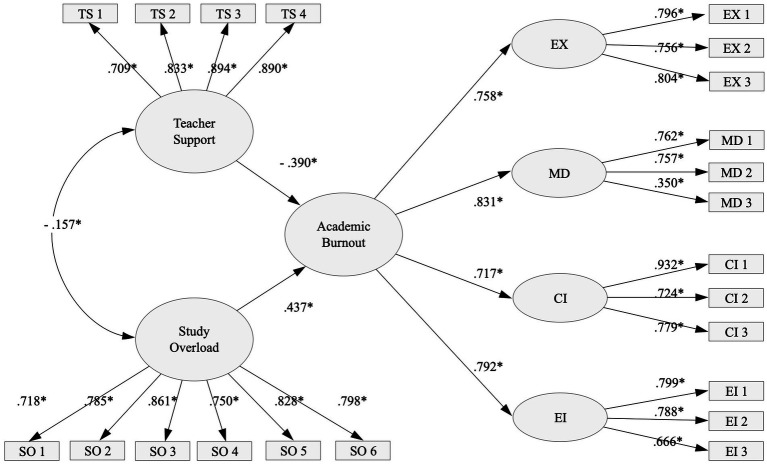
Graphical representation of the structural equation model between academic burnout, academic resources, and academic demands.

The SEM, based on previously described M4 model, obtains adequate fit indices: *χ*2 (201) = 543.509, *p* < 0.05; CFI = 0.938; TLI = 0.928; RMSEA = 0.061, 90% CI [0.055, 0.067]; SRMR = 0.061. Figure 1 shows that, as expected, teacher support and study overload are significantly negatively and positively related academic burnout, respectively.

Furthermore, according to Pearson’s correlation coefficient, teacher support and study overload are significantly related to academic burnout and its dimensions (see Table 3).

**Table 3:** Correlation analysis.

	Study overload	Teacher support	Exhaustion	Mental distance	Cognitive impairment	Emotional impairment
Exhaustion	0.462* [0.531, 0.387]	-0.315* [− 0.230, − 0.395]	-			
Mental distance	0.161* [0.249, 0.071]	-0.355* [− 0.272, − 0.432]	0.410* [0.331, 0.483]	-		
Cognitive impairment	0.290* [0.371, 0.204]	-0.289* [− 0.203, − 0.371]	0.440* [0.363, 0.511]	0.519* [0.449, 0.583]	-	
Emotional impairment	0.354* [0.432, 0.272]	-0.297* [− 0.212, − 0.378]	0.506* [0.435, 0.571]	0.458* [0.382, 0.527]	0.494* [0.422, 0.560]	-
Academic burnout	0.409* [0.482, 0.330]	-0.403* [− 0.324, − 0.477]	753.* [0.711, 0.790]	0.761* [0.719, 0.797]	0.789* [0.752, 0.822]	0.803* [0.768, 0.833]

* = *p* < 0.01; [] = 95% CI.

## Discussion

This brief report provides empirical evidence about the psychometric properties of the abbreviated version of the Burnout Assessment Tool for Students (BAT-S) in a sample of Chilean undergraduate students.

The obtained results show that the BAT-S performed well in a sample of Chilean undergraduate students showing acceptable reliability, which is consistent with previous studies in both academic (e.g., Popescu et al., 2023; Romano et al., 2022) and organizational settings (e.g., Schaufeli and De Witte, 2023; Vinueza-Solórzano et al., 2021). The internal structure of the Spanish version of the BAT-S is adequately explained by a model of four first-order factors (i.e., exhaustion, mental distance, cognitive impairment, and emotional impairment) and 1 second-order factor (i.e., academic burnout), which is compatible with the notion of a burnout syndrome. Moreover, this second-order model proves to be invariant to student’s gender, which is also consistent with previous studies (e.g., De Beer et al., 2020; Schaufeli et al., 2020; Schaufeli and De Witte, 2023; Sinval et al., 2022). In addition, criterion validity of the BAT-S was verified using the JD-R model, with an adequate fit of the proposed model and significant effects on academic burnout of both academic resources and demands, consistent with previous studies (e.g., De Beer et al., 2022b; Lesener et al., 2020; Salmela-Aro et al., 2022; Zeijen et al., 2024).

This study’s unique strength lies in its pioneering analysis of the psychometric properties of the BAT-S in a Spanish-speaking country, a novel and unexplored area of research. The findings of this research contribute to the initiation of a future research agenda related to academic burnout, starting with the conceptualization of BAT in countries where Spanish is an official language. Furthermore, our results suggest that the BAT-S may be adequately integrated into the JD-R model, which –as far as we know– has not been previously done in academic contexts. However, some limitations should be considered. First, the results should be cautiously generalized since our sampling does not represent Chilean students. Second, the data were collected using a cross-sectional self-reported survey instrument and may be prone to social desirability bias. Third, modification indices correlated two errors and improved the BAT-S′ fit. Notwithstanding these limitations, this study provides first evidence for using a brief tool that overcomes the theoretical and psychometric limitations of instruments traditionally used to measure academic burnout. Finally, according to the available literature from the organizational context, future research may consider analyzing cross-national representative samples (e.g., De Beer et al., 2020), establishing cut-off points for severe academic burnout (e.g., Schaufeli et al., 2023), to deepen the psychometric properties using alternative models (e.g., ESEM, Rasch analysis, and item-level analysis), and analyze the relationship with other academic demands (e.g., time pressure), academic resources (e.g., academic PsyCap), and academic outcomes (e.g., achievement) under de Study Demands-Resources theory (e.g., Bakker and Mostert, 2024).

## Data Availability

The raw data supporting the conclusions of this article will be made available by the authors, without undue reservation.

## Footnotes

^1^ https://burnoutassessmenttool.be
